# Polysaccharide from *Patinopecten yessoensis* Skirt Boosts Immune Response via Modulation of Gut Microbiota and Short-Chain Fatty Acids Metabolism in Mice

**DOI:** 10.3390/foods10102478

**Published:** 2021-10-16

**Authors:** Ying Li, Juan Qin, Yinghui Cheng, Yuqing Ai, Zhiyi Han, Meng Li, Yanxia Qi, Qiancheng Zhao, Zhibo Li

**Affiliations:** 1 College of Food Science and Engineering, Dalian Ocean University, Dalian 116023, China; liying@dlou.edu.cn (Y.L.); qinjuan202107@163.com (J.Q.); cheng-yh99@foxmail.com (Y.C.); 2531742233@163.com (Y.A.); zyzs14@163.com (Z.H.); limeng@dlou.edu.cn (M.L.); qiyanxia@dlou.edu.cn (Y.Q.); 2 Dalian Key Laboratory of Marine Bioactive Substances Development and High Value Utilization, Dalian Ocean University, Dalian 116023, China; 3 Liaoning Provincial Aquatic Products Analyzing, Testing and Processing Technology Scientific Service Centre, Dalian Ocean University, Dalian 116023, China; 4 Collaborative Innovation Center of Provincial and Ministerial Co-Construction for Marine Food Deep Processing, Dalian Polytechnic University, Dalian 116034, China; 5 Key Laboratory of Aquatic Product Processing and Utilization of Liaoning Province, Dalian Ocean University, Dalian 116023, China

**Keywords:** polysaccharides, scallop skirt, gut microbiota, short-chain fatty acids, immune response

## Abstract

Polysaccharide from marine shellfish has various bioactivities. In this study, the effects of polysaccharide from *Patinopecten yessoensis* skirt (PS) on boosting immune response in mice were evaluated, and the potential mechanisms were explored. The results showed that PS administration effectively increased the serum IgG and IgM levels, implying that PS had immune response-boosting properties. Moreover, PS administration could modulate the composition of the gut microbiota, and significantly improve short-chain fatty acids (SCFAs) metabolism, especially butyrate metabolism. Of note, the expression of the *Tlr2*, *Tlr7*, *MyD88*, *Tnfa,* and *Il1b* genes in toll-like receptor (TLR) signaling pathway was significantly increased. In summary, PS could boost immune response by modulating the gut microbiota and SCFAs metabolism correlating with the activation of the TLR signaling pathway. Therefore, PS can be developed as a special ingredient for functional product.

## 1. Introduction

Hypoimmunity has been considered as one of the principal manifestations of health problem due to stress and poor diet or rest [1]. In recent years, functional ingredients were found to have beneficial effects on reducing the risk of chronic diseases or metabolic syndrome [2,3,4]. Non-starch polysaccharides, a kind of prebiotics, can assist in the treatment or prevention of inflammatory bowel disease [5], restore immunity [6], modulate blood lipid [7], and inhibit tumor growth [8]. Studies have revealed that the biological effects of non-starch polysaccharides are linked to the modulation of the gut microbiota [9].

Shang et al. revealed the impact of non-starch polysaccharides on the metabolic activity of specific microbiota, immunity modulation and host health [10,11]. Trillions of microorganisms in the intestine are the most complex part of the host body and play a crucial role in nutrient metabolism, immune response, and overall health [12,13]. The gut microbiota is the core organ of polysaccharide metabolism in vivo. Non-starch polysaccharides are hard to be digested and absorbed by human body, but can serve as food by the gut microbiota during anaerobic fermentation [10,14]. Their metabolites, such as short-chain fatty acids (SCFAs), can provide nutrition to intestinal epithelial cells and stimulate the expression of immune related genes, which is crucial to produce host antibody and boost immune response [15,16]. In parallel, many studies suggested that polysaccharide could increase the composition of beneficial bacteria, thereby having a positive effect on bowel health [17].

Marine organisms are favorable sources of bioactive compounds with health-promoting effects [18]. Due to the special living environment, the synthesis process of polysaccharides in marine organisms is different from that of terrestrial organisms, and many bioactive compounds with novel structures and special functions have been produced [19]. Polysaccharide from *Sinonovacula constricta* had a strong effect of immunity modulation, which could enhance the capability of macrophage phagocytosis and the activities of immune-related enzymes [20]. The glycosaminoglycan from *Coelomactra antiquata* showed high anticoagulant titer and fibrinolytic value [21]. The sulfated polysaccharides from pacific abalone improved the obesity induced by high fat diet in mice through gut microbiota mediated pathway [22].

Scallops (*Patinopecten yessoensis*) have been cultured and consumed in the world [23]. Generally, live scallops have to be opened in raw by hand or heated by steaming to open the shell and obtain the edible parts of adductors. A large number of polysaccharide-rich by-products are produced during scallop processing, such as viscera and skirt. Polysaccharides from *P. yessoensis* viscera with high sulfate content and low molecular weight were reported to have anticoagulant properties [24]. Pyroptosis, oxidative stress, and inflammation were the main characteristics of inflammatory response caused by stimulated macrophages [25,26]. The extracts from scallops could inhibit the inflammatory response of macrophages [27]. However, to date, information on bioactivity of polysaccharide from *P. yessoensis* skirt (PS) is limited, and it remains uncertain whether PS can affect host immunity and the composition of the gut microbiota, which hinders further to develop value-added products from *P. yessoensis* skirt.

Therefore, the aims of this study were to assess immune response-boosting properties of PS by testing visceral indexes, serum IgG and IgM levels, and explore the potential mechanism through analyzing the diversity and composition of the gut microbiota, SCFAs metabolism, and the expression of immune-related genes.

## 2. Materials and Methods

### 2.1. Preparation of PS

Live scallops (*P. yessoensis*) were purchased from Zhangzidao Group Co., Ltd. (Dalian, China). Live scallops were washed and steamed at 100 °C for 5 min to open the shell, and the skirts were separated by hands. Then the skirts were cleaned and homogenized. The homogenate was extracted with distilled water at 90 °C for 4 h. The extracts were hydrolyzed by alkaline protease (Solarbio Science & Technology Co., Ltd., Beijing, China) and pronase (Solarbio Science & Technology Co., Ltd., Beijing, China), respectively. After enzymolysis and inactivation treatment at 90 °C for 15 min, the hydrolysate was centrifuged, and three volumes of 95% ethanol (Sinopharm Chemical Reagent Co., Ltd., Shanghai, China) were added to the supernatant. The mixture was centrifuged after standing overnight at 4 °C. After deproteinization with trichloroacetic acid (Sinopharm Chemical Reagent Co., Ltd., Shanghai, China), the precipitate was dialyzed with distilled water for 2 d and freeze-dried to obtain PS. The chemical characterization of PS was in supplemental data (Table S1. Chemical characterization of PS) including essential component, monosaccharide composition, and molecular weight.

### 2.2. Digestibility of PS by Artificial Human Saliva and Gastric Acid

The digestion by artificial human saliva and gastric acid was conducted according to Yu’s method [3]. Briefly, the pH of hydrochloric acid buffer was adjusted to 1.0, 2.0, 3.0, 4.0, and 5.0. Then, 100 mg PS was dissolved in the hydrochloric acid buffer and cultured at 37 °C for 8 h. The reducing sugar content was measured at 0, 2, and 4 h by the 3,5-dinitrosalicylic acid method, and the total sugar contents were measured at 0 h by the phenol sulfuric acid method [28]. The indigestibility was calculated as follows:

Hydrolysis degree (%) = (R_1_ − R_0_)/(T_0_ − R_0_) × 100% (1)

where R_1_ and R_0_ are the reducing sugar content at some point and the initial reducing sugar content, respectively. T_0_ was the initial total sugar content.

### 2.3. Animal Experiment

All experiments were performed in compliance with the Chinese National Standard: Laboratory Animals-Guideline for ethical management of animal welfare and approved by the Institutional Animal Care and Use Committee of Weitong Lihua Laboratory Animal Technology Co., Ltd. (P2020046, Beijing, China).

Animals: four-week-old male SPF C57BL/6 mice were purchased from Weitong Lihua Laboratory Animal Technology Co., Ltd. (Beijing, China). All mice were raised at 25 ± 1 °C with a 12 h light/dark cycle and had access to food and water ad libitum.

Feeding process: Twenty-seven mice were equally divided into three groups for gavage and reared for two weeks after a one-week acclimatization period. The groups were shown in Table 1. Every day, group C was given a gavage of 0.35 mL of distilled water, group L was given a gavage of 0.35 mL of PS (equivalent to 50 mg of polysaccharide/kg body weight), group H was given a gavage of 0.35 mL of PS (equivalent to 100 mg of polysaccharide/kg body weight), and the feeding period was 14 days. After 14 days, water and feed were removed for 8 h, and then, the mice were killed.

### 2.4. Sample Collection

The mice were weighed every other day. The feces of the mice were collected on day 0, day 7, and day 14 for bacterial community analysis. On the 14th day, the mice were sacrificed, and the samples of serum, spleen, thymus, gut contents and gut were collected. The spleen and thymus were weighted to evaluate the immune organ indexes. The serum, gut contents and gut were stored at −80 °C until detection.

### 2.5. Immune Index

The immune organ indexes were determined by the ratio of the weight of spleen or thymus to the weight of the mice. The IgG and IgM content in serum were followed the protocol of the Mouse IgG ELISA Kit and IgM ELISA Kit (Lanpai Biotechnology Co., Ltd., Shanghai, China).

### 2.6. Gut Microbiota Analysis

The collected feces were transferred to liquid nitrogen for preservation. Total DNA was extracted from fecal samples by using a Bacterial DNA Kit (Tiangen Biotech Co., Ltd., Beijing, China) according to the manufacturer’s instructions. The V3-V4 region of the bacterial 16S rRNA gene was amplified according to protocols described by Liu’s work [29]. The 16S amplicons were then sent to Novogene Co., Ltd. (Tianjin, China) for sequencing and analysis of the bacterial community. The library preparation and sequencing methods were followed Liu’s work [29].

UParse (Uparse v7.0.1001, http://www.drive5.com/uparse/ (assessed on 15 July 2021).) was used to cluster all the effective tags of all samples, and the sequence was clustered into operational taxonomic units (OTUs) with 97% identity. For OTUs, the SSUrRNA database of SILVA132 (http://www.arb-silva.de/ (assessed on 15 July 2021)) was used for species annotation analysis, and the community composition of each sample was counted. Qiime software (Version 1.9.1), (https://qiime2.org/ (assessed on 15 July 2021)) was used to calculate the observed species, Shannon and ACE. The R software was used to analyze the differences of Beta diversity index between groups. Alpha diversity was evaluated by the observed species, ACE and Shannon indexes, and beta diversity was evaluated by principal coordinate analysis (PCoA) based on unweighted UniFrac distance. Linear discriminant analysis coupled with effect size (LEfSe) analysis was performed by LEfSe software (http://huttenhower.sph.harvard.edu/galaxy/ (assessed on 15 July 2021)), and the filter value of Linear Discriminant Analysis Score was set as 4 by default.

### 2.7. Determination of SCFA Contents

Fecal samples were collected and added to 50 μL of 15% phosphoric acid (Aladdin Biochemical Technology Co., Ltd., Shanghai, China) on the 14th day. Then, 100 μL of a 125 μg/mL internal standard (isohexanoic acid, Aladdin Biochemical Technology Co., Ltd., Shanghai, China) solution and 400 μL of diethyl ether (Aladdin Biochemical Technology Co., Ltd., Shanghai, China) were added, and the samples were homogenized for 1 min. The supernatant was collected for testing after centrifugation at 12,000 rpm and 4 °C for 10 min. Thermo TRACE 1310-ISQ GC-MS (Thermo Fisher Scientific Inc., Waltham, MA, USA) equipped with an Agilent HP-INNOAX (Agilent Technologies, Co., Ltd., Beijing, China) capillary column (30 m × 0.25 mm ID × 0.25 μm) was used to determine the SCFA contents.

Chromatographic conditions included the following: injection volume, 1 μL; shunt ratio, 10:1; inlet temperature, 250 °C; ion source temperature, 230 °C; transmission line temperature, 250 °C; quadrupole temperature, 150 °C; program with an initial temperature of 90 °C, 90–120 °C at 10 °C/min, 120–150 °C at 5 °C/min, and then 150–250 °C at 25 °C/min maintained for 2 min; carrier gas, helium; and carrier gas flow rate, 1.0 mL/min.

MS conditions included the following: electron bombardment ionization (EI) source; SIM scanning mode; and electron energy, 70 eV.

### 2.8. Intestinal Immune Related Gene Expression

Intestinal immune-related gene expression was evaluated using qRT-PCR according to Liu’s method [30]. Extraction of the total RNA of the intestinal tissue, cDNA synthesis and RT-PCR were performed using an RNAprep Pure Tissue Kit (Tiangen Biotech Co., Ltd., Beijing, China), TransScript Reverse Transcriptase (TransGen Biotech Co., Ltd., Beijing, China) and TransStart Top Green qPCR SuperMix (TransGen Biotech Co., Ltd., Beijing, China), respectively. The primers used in this study are listed in Table 2. The results were analyzed using the 2^−ΔΔCt^ method, and the housekeeping gene *Actb* was used as an internal reference to normalize the data.

### 2.9. Statistical Analyses

Each sample was analyzed in triplicate. The data were expressed as the average ± SD. Variance analysis and *t* tests were used to determine the significance of the differences between two samples at a level of *p* < 0.05, and the *t* test was replaced by a one-way ANOVA when comparing the differences of multiple samples.

## 3. Results

### 3.1. Digestibility of PS

**Figure 1:**
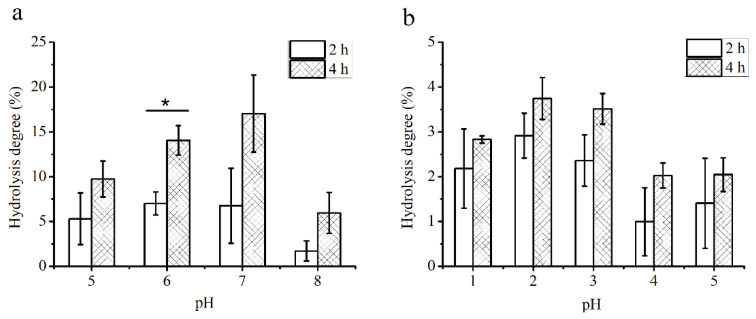
Hydrolysis degree of PS in artificial human saliva (**a**) and in a simulated gastric acid (**b**). * Indicates that there is a significant difference between treated times (*p* < 0.05).

The digestibility of PS by artificial saliva and gastric acid was shown in Figure 1. The constituents of human saliva are similar, but the range of pH is different. The results showed that the hydrolysis degree of PS increased with hydrolysis time at the same pH ranged from 5 to 8, and the hydrolysis degree of PS after 4 h was significantly higher than that after 2 h at pH 6. Besides, the results also indicated that the hydrolysis degree of PS increased to the highest of 17.03% at pH 7 after 4 h, but no significant difference was observed among pH from 5 to 7 at the same hydrolysis time (Figure 1a).

Furthermore, the ability of PS to resist degradation by gastric acid was tested. The pH of gastric juice ranged from 1 to 3, and food stayed in the stomach for approximately 4 h [3]. The hydrolysis degrees of PS increased with hydrolysis time at the same pH from 1 to 5, but there was no statistically significant difference (Figure 1b). After 4 h, the hydrolysis degree of PS reached to 3.74% at pH 2. However, the hydrolysis degree of PS showed no significant difference among pH 1 to 5 at the same hydrolysis time. The results indicated that PS was quite stable in an acidic environment and had good resistance to α-amylase hydrolysis.

### 3.2. Effect of PS on the Immune Indexes

The thymus provides the differentiation and maturation T cells for peripheral immune organs, while the spleen is the site of immune response and immunoglobulin production. Thymus and spleen are important immune organs in human body, thus, the thymus and spleen index are considered to be able to reflect the strength of immune function [31]. In the study of the relationship between polysaccharide and immunity, many researchers found that after administration with polysaccharides, there was no significant difference in thymus index, but the spleen index increased significantly [32,33,34]. Similar to previous results, after 14 d of feeding, there was no significant difference in body weight (data not shown) or thymus index among all groups, but the spleen index of group H14 was significantly higher than that of group C14 (Table 3). In addition, as shown in Table 3, the effects of PS on the contents of IgG and IgM in serum were assessed. The results showed that the levels of IgG and IgM in groups L14 were significantly higher than those in group C14, and group H14 showed the highest level among the groups. This result suggested that PS had an immunopotentiative effect by increasing the content of immunoglobulin in serum.

### 3.3. Microbial Community Diversity in the Mice Gut

**Figure 2:**
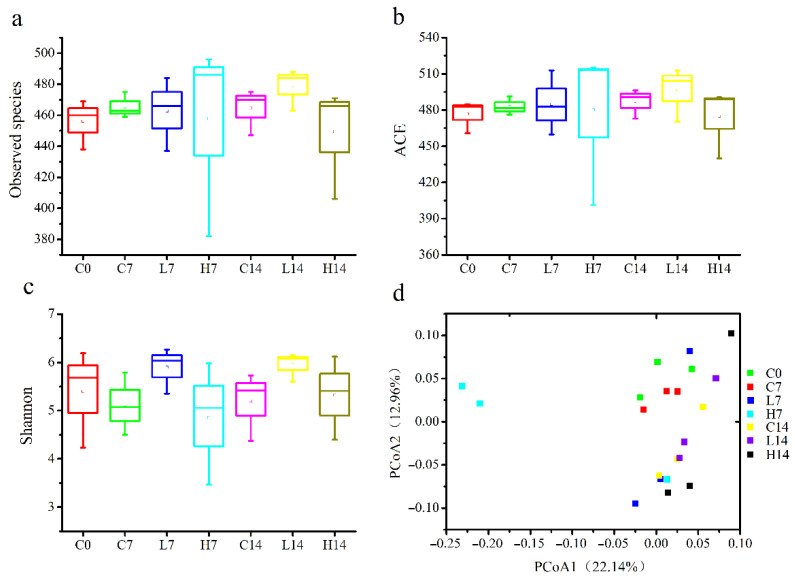
Alpha and beta diversity of the gut microbiota in mice during administration of PS. (**a**) Observed species, (**b**) ACE estimate of richness, (**c**) Shannon estimate of richness values, and (**d**) PCoA (the linear distance in (**d**) between the two samples represents the difference in microbiota.). C0 indicates the samples from groups C, L, and H at day 0; C7 and C14 indicate the samples from the group C at days 7 and 14, respectively; L7 and L14 indicate the samples from group L at days 7 and 14, respectively; and H7 and H14 indicate the samples from group H at days 7 and 14, respectively.

The gut microbiota has long been considered as the key to health homeostasis and the treatment for numerous diseases [35]. Accumulating studies have revealed that the decrease of gut microbiota diversity was a risk factor of various metabolic diseases [36]. It is necessary to study whether PS can enhance immune response by modulating the gut microbiota. The observed species (Figure 2a) and ACE indexes (Figure 2b) were used to reflect the community richness, and Shannon indexes (Figure 2c) were used to represent the community diversity among different groups. The results showed that no significant differences were detected among the groups in terms of the observed species, ACE and Shannon indexes, implying that during the two-week experiment, PS could maintain the community richness and diversity of the gut microbiota.

The overall structural changes between groups were analyzed by PCoA (Figure 2d). On day seven, there was a significant separation in the microbial community composition between the group H7 and group C7. However, no separation was observed at day 14. This result indicated that short-term and high-dose administration with PS caused significant differences in the gut microbiota structure and led to a significant difference in beta-diversity. However, after long-term administration, the gut microbiota might adapt to PS, and the mice stabilized the gut microbiota through self-regulation.

### 3.4. Changes in Gut Microbiota

**Figure 3:**
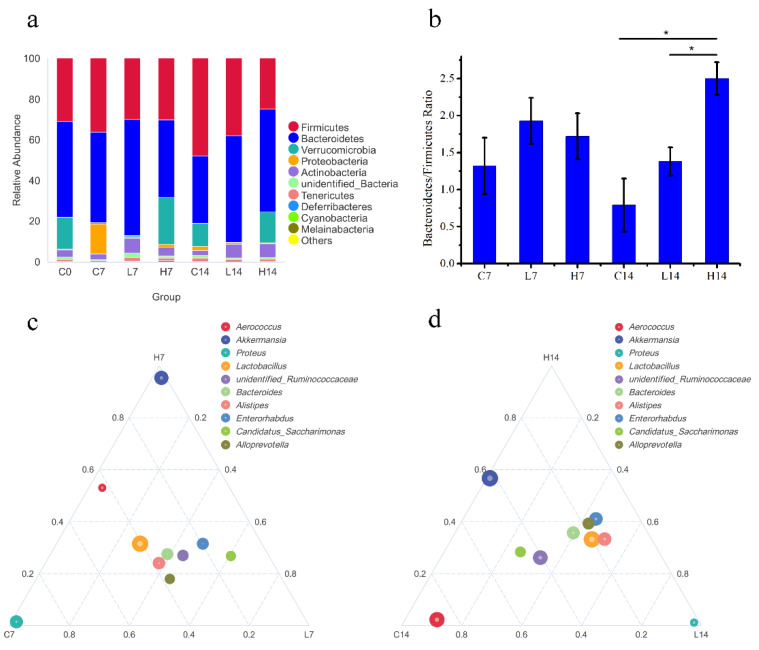
Composition and distribution of the gut microbiota in mice. (**a**) Changes in the relative abundance of the top 10 phyla (on days 0, 7 and 14). (**b**) The ratio of Bacteroidetes to Firmicutes in different groups. A significant difference compared to group C14 is represented as *. (**c**) Ternary plot at the genus level on day seven. (**d**) Ternary plot at the genus level on day 14. The size of the circle is proportional to the relative abundance. The closer the circle is to a vertex, the higher the abundance of the genus in this group.

To further elucidate the structural response of gut microbiota after PS administration, the relative abundance of the top 10 species at the phylum level in each group was analyzed (Figure 3a). Despite the consistency of the gut microbiota structure, some differences in their relative abundance were observed. Bacteroides and Firmicutes were the dominant gut bacteria in all the groups. On day seven, the ratio of Bacteroidetes to Firmicutes showed no significant difference among all groups (Figure 3b). However, on day 14, the ratio of Bacteroidetes to Firmicutes in group H14 was significantly increased compared with that in group C14 and L14.

To further clarify the differences in dominant species in three groups (C, L, and H), the genus with the highest average abundance was selected to generate a ternary phase diagram (Figure 3c,d). On day seven, compared with those in group C7, the abundances of *Enterorhabdus*, *Candidatus*_*Saccharimonas*, *Ruminococcaceae*, and *Alloprevotella* increased in group L7, and the abundance of *Akkermansia* increased in group H7. However, the abundance of *Proteus* reduced in both groups L7 and H7. On day 14, compared with those in group C14, the abundances of *Lactobacillus*, *Enterorhabdus*, *Alloprevotella*, and *Alistipes* increased in groups L14 and H14, while the abundance of *Akkermansia* reduced in group L14. The abundance of *Aerococcus* in group C14 was higher than that in groups L14 and H14.

**Figure 4:**
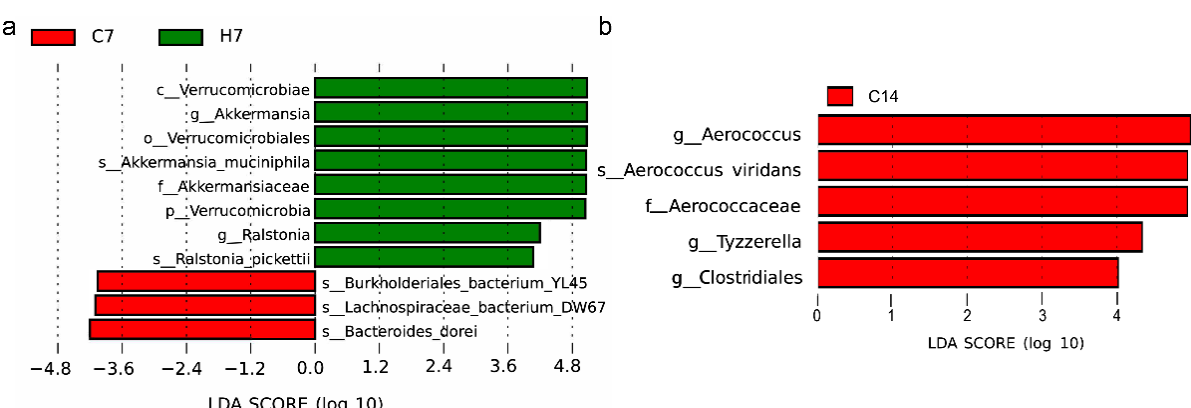
LEfSe of taxa with significantly different abundances in different groups. (**a**) C7 vs. H7; (**b**) C14 vs. H14.

LEfSe was applied to further identify the most differentially abundant taxons between the control group and the administration groups at the OTU level using LDA. Figure 4 showed the bacteria abundance with significant differences in different groups. The results revealed that, compared with those in group C7, *Akkermansia* and *Ralstonia* in group H7 were the predominant communities. At the genus levels, group C14 showed higher abundance of *Aerococcus* than group H14, along with greater abundance of *Tyzzerella* and *Clostridiales*.

### 3.5. SCFA Content

**Figure 5:**
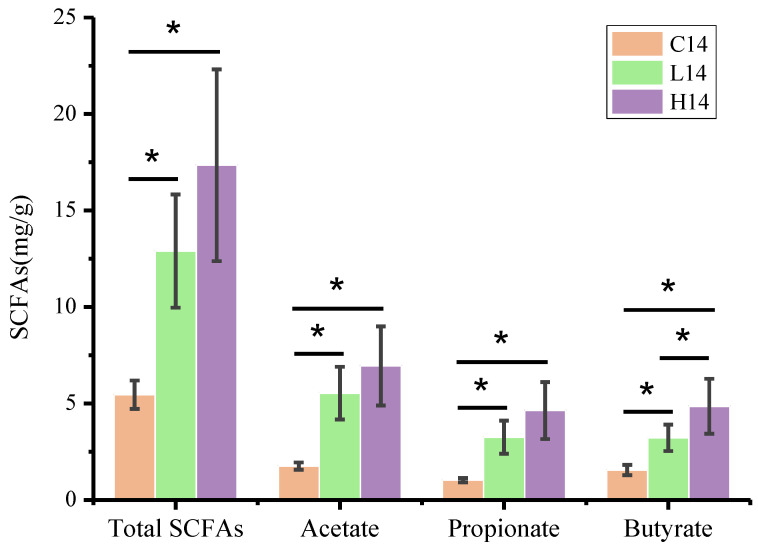
Effect of PS on the SCFA concentration in feces. * Indicates that there was significant difference between groups (*p* < 0.05).

SCFAs are a series of metabolites of non-starch carbohydrates fermented by the gut microbiota that can provide nutrients for the host and have beneficial effects on the host health [37,38]. In this study, the SCFA contents in feces were determined on day 14 as shown in Figure 5. The results showed that the total SCFA contents in groups L14 and H14 were significantly higher than those in group C14, and the contents of acetate, propionate and butyrate showed the same trend as the total SCFA contents. In addition, the butyrate content in group H14 was significantly higher than that in group L14 and group C14. SCFAs, especially, butyrate, play a key role in the interaction between the gut microbiota and the activation of the immune system. The results suggested that PS could significantly increase the content of SCFAs in the feces of mice, thus helping host improve their immunity, and the reason might be related to the fermentation of the PS [39].

### 3.6. Immune-Related Gene Expression

**Figure 6:**
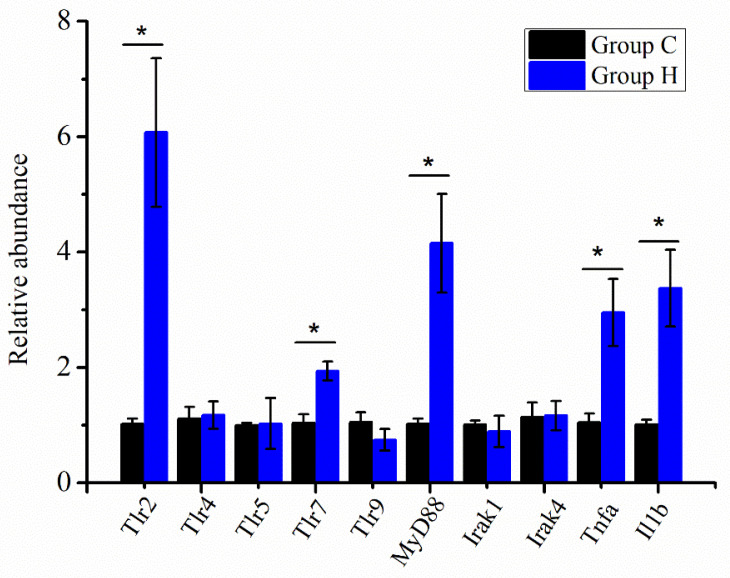
Expression of immune-related genes in intestinal tissue on day 14. The results were analyzed using the 2^−ΔΔCt^ method, and the 2^–ΔΔCt^ of genes in the group C was set as 1. * Indicates that there was significant difference between groups (*p* < 0.05).

The relative expression of key genes was detected in the Toll-like receptor (TLR) signaling pathway in intestinal tissues to study the potential mechanisms of PS administration on immune response. As shown in Figure 6, the relative expression of *Tlr2*, *Tlr7*, *MyD88*, *Tnfa*, and *Il1b* in group H14 was significantly higher than that in group C14. However, the expression of other genes showed no difference between the two groups. In addition, the relative expression of all genes in TLR signaling pathway was no significant difference between group L and group C (data not shown). These results suggested that PS initiated immune response by activating the TLR signaling pathway. In group H14, the increased expression of inflammatory cytokines such as *Tnfa* and *Il1b* indicated that PS positively regulated the immune activity by increasing the secretion of cytokines such as TNF-α and IL-1β and promoted the maintenance of intestinal mucosal immune homeostasis.

## 4. Discussion

In recent years, with continuous in-depth research, increasing evidence has linked the body’s immune system with the gut microbiota [40,41,42]. In this study, the diversity of gut microbiota remained stable after PS administration without significant changes. It is now widely believed that gut microbiota stability is an important parameter in host–microbe symbiosis, and plays a key role in human health [36]. Similar to other studies, the gut microbiota in this study (Figure 3a) was dominated by Firmicutes and Bacteroides [43,44]. After 14 days of feeding, the ratio of Bacteroidetes to Firmicutes of group H14 was significantly higher than that of group C14 (Figure 3b). The ratio of Bacteroidetes to Firmicutes is usually related to health conditions, such as obesity and allergies, and healthy bodies tend to show an elevated ratio [45,46,47].

As shown in Figure 3c,d, at the beginning of the feeding time, Verrucomicrobia was found in the flora, and the main genus was *Akkermansia*. With the extension of feeding time, *Akkermansia* abundance in groups C and L decreased, while it remained constant in group H. *Akkermansia* can produce propionic acid, which acts on intestinal tissue through Gpr43, thus causing a series of changes in signaling pathways to achieve immune regulation [48]. Derrien et al. colonized sterile mice with *Akkermansia* and found that the gene expression changes in the host intestinal tissue were mainly focused on genes related to the immune response [49]. This result suggested that *Akkermansia* regulated the body’s metabolic balance and immune tolerance. In terms of immune indexes, only the spleen index of group H14 significantly increased, and the concentrations of serum IgG and IgM in the high-dose groups were significantly higher than those in the other groups (Table 3). The increase of IgG and IgM boosts the immune response of the body, but may also have a partial negative effect on specific populations, such as patients with autoimmune diseases and multiple myeloma [50].

On day seven, the relative abundance of *Proteus* in group G increased significantly, while that in the other groups remained unchanged (Figure 3c). *Proteus* has been associated with ulcerative lesions in the gastrointestinal tract of immunodeficient mice [51]. It was suggested that PS decreased the abundance of *Proteus*, and had indirectly protective effect on the intestinal tract of mice. On day 14, the relative abundance of *Aerococcus* in group C14 was significantly higher than the other groups (Figure 3d). *Aerococcus* has been an increasingly acknowledged human pathogen [52]. However, as opportunistic pathogens, *Proteus* and *Aerococcus* are generally considered environmental organisms and can be found in the human and animal gastrointestinal tract, skin, and oral mucosa, as well as in feces, soil, water, and plants [53,54]. Therefore, the increase in *Proteus* and *Aerococcus* abundance may increase the risk of disease under specific conditions, and PS intake can reduce this risk.

SCFAs are produced by a subset of microbes in the gut microbiota through the fermentation of indigestible polysaccharides [37], and they play important roles in immune regulation. SCFAs regulate gene expression in epithelial barrier cells, innate immune cells, and antigen-specific adaptive immunity mediated by T and B cells [55]. In this study, PS administration significantly increased the content of SCFAs in mice feces, especially butyrate (Figure 5), which might be the result of PS degradation by gut microorganisms [56]. Butyrate can directly affect the growth and differentiation of colon cells, and it is the main energy source of colon cells, and can enhance intestinal barrier function and mucosal immunity [57,58]. The butyrate content in group H14 was significantly higher than that in groups C14 and L14, but no significant difference was observed in the abundance of butyrate-producing bacteria such as *Ruminococcaceae* and *Lachnospiraceae.* This result suggested that the increase of butyrate content in the feces of mice administration with PS was not caused by the increase of butyrate producing bacteria abundance, but it may result from the fermentation of PS. The significant increase in SCFA contents in feces after PS administration might be an important reason for immunoenhancement.

The gut microbiota not only produces a wide range of immune barriers but also stimulates the development of the host immune system and the occurrence of cellular immunity. Therefore, the gut microbiota often plays a role in regulating immune-related signaling pathways, such as the TLR, MAPK, and NF-κb signaling pathways [59,60]. The TLR signaling pathway is important in the innate immune system and immune responses. Stimulation of *Tlr2* and *Tlr4* can lead to an increase in two signal components, *MyD88* and *Irak*, and subsequently mediate the activation of *NF-κB* [61]. After detecting the expression of TLR signaling pathway genes in the intestinal tissue, it was found that the *Tlr2*, *Tlr7*, and *MyD88* genes of mice with PS administration were significantly upregulated, which was accompanied by an increase in the levels of inflammatory cytokines, such as TNF-α and IL-1β (Figure 6). The production of inflammatory cytokines amplifies the immune response, but it also carries the risk of chronic inflammatory diseases and autoimmune diseases [62].

**Figure 7:**
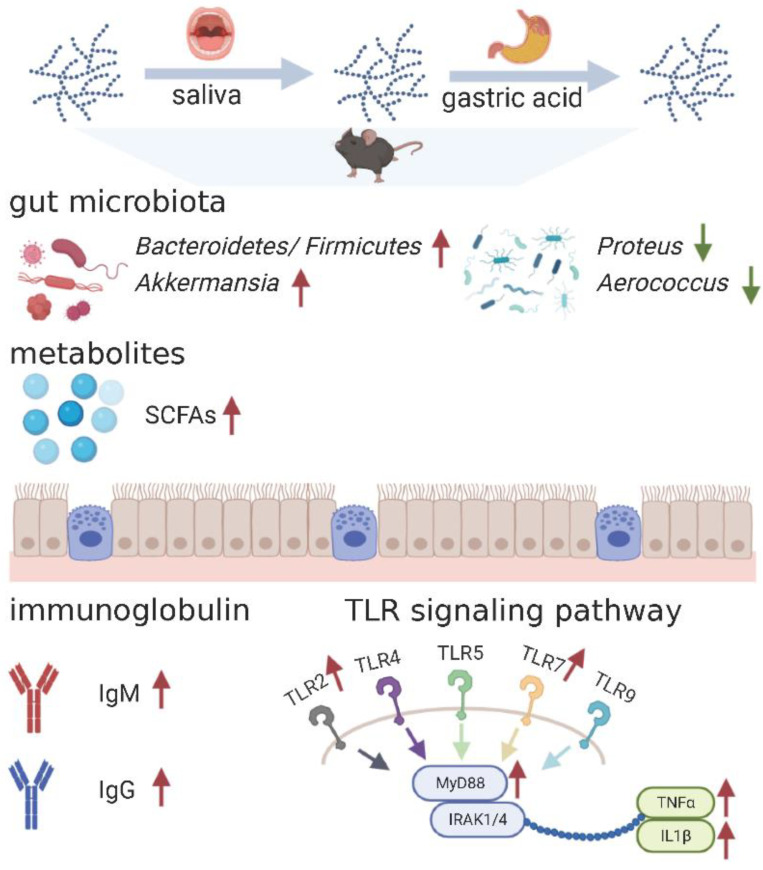
Potential mechanisms of the effects of PS on immune response. Red arrow represents up regulation; Green arrow represents down regulation; SCFAs: short-chain fatty acid. This figure was created with icons provided by bio render (https://biorender.com (assessed on 10 October, 2021)).

In conclusion, as shown in Figure 7, these results suggested that changes in the gut microbiota and SCFAs contents caused by PS administration stimulated the immune response of mice, accompanied by an increase in immune parameters (thymus and spleen index, IgG and IgM), and immune-related gene (*Tlr2*, *Tlr7*, *MyD88*, *Tnfa*, and *Il1b*) expression. Therefore, PS could be a potential immune-enhancing bioactive ingredient for functional product development.

## Figures and Tables

**Table 1:** Experimental groups.

Group	Characterization
C	Control group, daily gavage of 0.35 mL water
L	Low-dose administration group, daily gavage of equivalent to 50 mg polysaccharides/kg body weight
H	High-dose administration group, daily gavage of equivalent to 100 mg polysaccharides/kg body weight

**Table 2:** Primers used in this study.

Gene	Primers (5′–3′)
*Tlr2*	forward: GACTCTTCACTTAAGCGAGTCT reverse: AACCTGGCCAAGTTAGTATCTC
*Tlr4*	forward: GCCATCATTATGAGTGCCAATT reverse: AGGGATAAGAACGCTGAGAATT
*Tlr5*	forward: GCTTCGTGTTTTGGACATAACT reverse: GGTGGATATGTTGTAGAGGGAG
*Tlr7*	forward: TGTGATGCTGTGTGGTTTGTCTGG reverse: TTTGACCTTTGTGTGCTCCTGGAC
*Tlr9*	forward: GATCTGCCCAAACTCCACACTCTG reverse: TCTGACAAGTCCACAAAGCGAAGG
*MyD88*	forward: CGGAACTTTTCGATGCCTTTAT reverse: CACACACAACTTAAGCCGATAG
*Irak1*	forward: GTTATGTGCCGCTTCTACAAAG reverse: GATGTGAACGAGGTCAGCTAC
*Irak4*	forward: CTTCGGCGTGGTTCTGTTGGAG reverse: CCGCATCGCTCATCTTCTCATCC
*Tnfa*	forward: ATGTCTCAGCCTCTTCTCATTC reverse: GCTTGTCACTCGAATTTTGAGA
*Il1b*	forward: TCGCAGCAGCACATCAACAAGAG reverse: AGGTCCACGGGAAAGACACAGG
*Actb*	forward: CTACCTCATGAAGATCCTGACC reverse: CACAGCTTCTCTTTGATGTCAC

**Table 3:** Immune parameters of each group after 14 days of feeding.

Parameters	C14	L14	H14
Spleen index (mg/g)	2.77 ± 0.16 ^a^	2.87 ± 0.16 ^a,b^	3.33 ± 0.53 ^b^
Thymus index (mg/g)	4.83 ± 0.16 ^a^	5.06 ± 0.43 ^a^	5.52 ± 0.80 ^a^
IgG (g/L)	15.82 ± 0.96 ^a^	19.99 ± 1.18 ^b^	25.86 ± 1.40 ^c^
IgM (g/L)	2.24 ± 0.12 ^a^	2.86 ± 0.28 ^b^	3.72 ± 0.09 ^c^

^a–c^ In the same line, the data with the same letter indicates that the difference is not significant (*p* > 0.05).

## Data Availability

The data presented in this study are available on request from the corresponding author.

## Supplementary Materials

The following are available online at https://www.mdpi.com/article/10.3390/foods10102478/s1, Table S1: Chemical characterization of PS.
