# Supplementary material for: Polysaccharide from Patinopecten yessoensis Skirt Boosts Immune Response via Modulation of Gut Microbiota and Short-Chain Fatty Acids Metabolism in Mice
Source: Foods. 2021 Oct 16;10(10):2478. doi: 10.3390/foods10102478 (PMC8535924; doi:10.3390/foods10102478)
Supplement: Supplementary file 1 [file foods-10-02478-s001.zip › foods-1403388-supplementary.pdf]

Table S1. Chemical characterization of PS

Content	Parameters	Value
Essential component (%)	moisture	11.12±0.87
	ash content	5.08+0.05
	protein	19.48+2.04
	total sugar	61.32±1.30
	reducing sugar	0.89±0.03
Mass ratio of monosaccharides	mannose	3.36
	glucosamine	39.44
	galactosamine	9.72
	glucose	20.85
	galactose	26.63
Molecular weight (Da)		2.538×10^5
